# Reduced amplitude of low frequency fluctuation in the sensorimotor cortex in early psychosis: association with motor dexterity and hyperconnectivity

**DOI:** 10.1038/s41537-026-00789-0

**Published:** 2026-07-29

**Authors:** Jason Smucny, Korey P. Wylie, Jason R. Tregellas, Stephanie M. Hare

**Affiliations:** 1 Department of Psychiatry and Behavioral Sciences, University of California, Davis, CA USA; 2 Department of Psychiatry, Anschutz Medical Campus, University of Colorado School of Medicine, Aurora, CO USA; 3 Research Service, Rocky Mountain Regional VA Medical Center, Aurora, USA CO; 4 Department of Psychiatry, Maryland Psychiatric Research Center, University of Maryland School of Medicine, Baltimore, MD USA

**Keywords:** Schizophrenia, Psychosis, Neural circuits

## Abstract

Previous studies have demonstrated widespread regional abnormalities in blood oxygen level-dependent (BOLD) amplitude of low frequency fluctuation (ALFF) in psychotic disorders. How regional ALFF is related to functional connectivity and behavior in psychotic illness, however, is poorly understood. Here we examined voxelwise ALFF across the brains of subjects with early psychosis (EP) vs. healthy controls (HCs) as well as its relationships to functional connectivity and task performance. Resting state fMRI data from 55 HCs and 109 participants with EP from the Human Connectome Project—Early Psychosis were analyzed. Our analytic strategy was to examine ALFF across the whole brain to determine what regions showed significant differences in the psychosis group and then determine how those regional abnormalities in ALFF were related to connectivity patterns with other brain areas. We then examined correlations between these measures and performance on task-relevant to the observed circuitry. Significantly lower ALFF was observed in the sensorimotor cortex in participants with EP vs. HC. Decreased sensorimotor ALFF in EP, furthermore, was associated with poor motor dexterity and cognitive function. Finally, in participants with EP, ALFF showed increased connectivity with the cerebellum, anterior cingulate, and insula. Our results suggest that spontaneous low-frequency blood oxygen level-dependent fluctuation amplitude is significantly lower in sensorimotor cortex in EP, and that this change is associated with poor motor skills and cognition. Hyperconnectivity within the observed circuits in participants with EP may indicate more widespread pathology with areas that show abnormally low levels of baseline BOLD fluctuation.

## Introduction

Schizophrenia and other psychotic disorders are associated with prominent abnormalities in intrinsic brain function. One powerful method of assessing the brain’s intrinsic function is to examine its amplitude of low frequency fluctuation (ALFF) during resting state functional magnetic resonance imaging (fMRI). At each voxel, ALFF measures the total power within the low-frequency band (typically between ~0.01 and 0.1 Hz) of the blood-oxygen-level-dependent (BOLD) signal and thus provides a readout of fluctuations in intrinsic brain metabolic activity that are associated with neuronal function^1^.

Interestingly, previous studies in psychotic disorders have reported widespread differences in regional ALFF. The first ALFF paper in schizophrenia found reduced low frequency BOLD amplitude in the lingual gyrus, cuneus, and precuneus, and increased amplitude in the parahippocampal gyrus^2^. Subsequent studies found ALFF abnormalities across a range of regions. First episode schizophrenia was associated with decreased ALFF in the bilateral inferior parietal gyri, right precuneus, and left medial prefrontal cortex as well as increased ALFF in the bilateral putamen and bilateral occipital gyrus in a meta-analysis of 13 studies^3^. Chronic schizophrenia was associated with decreased ALFF in the bilateral postcentral gyrus, left precuneus and right occipital gyrus as well as increased ALFF in the bilateral inferior frontal gyri, bilateral superior frontal gyrus, left amygdala, left inferior temporal gyrus, right anterior cingulate cortex and left insula in 15 studies^3^. A 2024 meta-analysis also reported decreased ALFF in the postcentral gyrus, posterior cingulate and precuneus as well as increased ALFF in the inferior frontal gyrus and striatum^4^.

Although these meta-analyses suggest ALFF is altered in several regions in schizophrenia, knowledge gaps remain. First, the relationships between ALFF and behavior/cognitive function in psychosis are poorly understood, as most ALFF studies in psychotic illness either do not examine or report on behavioral associations. Notable exceptions include work by Fryer et al.^5^, who found associations between working memory and fractional ALFF (fALFF, or ALFF normalized to the entire scan frequency range) in frontoparietal executive regions in chronic schizophrenia, and Wang et al.^6^, who reported negative correlations between ALFF in the somatosensory cortex and processing speed across patients with first episode and chronic schizophrenia. Second, how local differences in ALFF affect functional connectivity patterns across the brain is unclear, particularly in psychosis. Limited work in other disorders, such as major depression and bipolar disorder, suggests that regions showing ALFF differences also differ in their connectivity patterns to other brain areas^7,8^. It is currently unclear whether participants with EP would show a similar pattern of findings. Finally, to our knowledge, an analysis of ALFF has not yet been conducted using data from the Human Connectome Project – Early Psychosis (HCP-EP). Using imaging protocols based on the original HCP, the HCP-EP includes high quality imaging, behavioral, clinical, and neurocognitive data in participants with affective and non-affective early psychosis (within the first 3 years of the onset of psychotic symptoms)^9^. Advantages of HCP-EP data include its robust sample size (*n* > 100 participants with EP and >50 healthy controls (HCs), harmonization of imaging procedures across its 3 identical scanners, comprehensive clinical and cognitive battery, and recruitment of EP individuals (before chronic effects of antipsychotic medication and/or illness are apparent in the brain).

The three aims of this study were: (1) to conduct a whole-brain analysis comparing regional ALFF in EP vs. HCs in the HCP-EP dataset to identify cluster(s) with significantly different ALFF in EP(we also examined fALFF to determine if normalizing ALFF signal across the analyzed frequency range affected results). We then (2) examined significant group differences in connectivity with clusters of abnormal ALFF by performing voxelwise seed-based connectivity analysis (in the manner of^10^). Finally, we (3) examined whether and how the extracted low-frequency power in the identified ALFF clusters, as well as ALFF cluster-defined, seed-based connectivity patterns were associated with relevant behavioral/cognitive task performance. For the last aim, we included tasks typically recruited by that region (for example, if our ALFF analysis found group differences in the dorsolateral prefrontal cortex, we would examine relationships with working memory task performance (as in Fryer et al.^5^)) as well as overall cognition (as an exploratory measure).

## Results

### Demographic, clinical, behavioral, and fMRI movement information

Demographic and clinical information for the sample are presented in Table 1. Briefly, the EP group was significantly younger but did not show differences in sex or handedness distribution vs. HCs. As demonstrated previously^11^, the EP group performed significantly worse than HCs on the pegboard task as demonstrated by significantly lower scores in EPs for both the dominant and non-dominant hand. No relationships were observed between site and pegboard performance. The proportion of valid scans was 99% for controls and 97% for EP individuals, and participants with EP moved more than controls (Table 1). fMRI data from one HC and 10 EP individuals were excluded for not meeting fMRI quality control criteria (see “Methods”).

**Table 1:** Demographic and clinical information.

Measure	Healthy Control Group (*n* = 55)	Early Psychosis Group (*n* = 109)	*t* or *χ*^2^	*p*
Age	24.82 (4.070)	22.89 (3.76)	3.02	0.003
Sex M/F	36/19	66/43	0.37	0.54
Handedness R/L/Ambi	44/10/1	95/9/4	3.73	0.16
Site location	8/24/10/13	19/52/9/29	3.55	0.31
*N* affective/non-affective psychosis	–	28/81	–	–
PANSS total score	–	48.95 (10.56)	–	–
PANSS positive score	–	10.88 (3.82)	–	–
PANSS negative score	–	12.42 (4.94)	–	–
PANSS disorganization score		11.74 (3.63)	–	–
PANSS motor retardation score	–	1.44 (0.92)	–	–
Taking antipsychotic medication Y/N	–	43/65	–	–
Current antipsychotic dose (chlorpromazine equivalent mg/day)	–	377.91 (202.75)	–	–
Pegboard age-corrected standard score, dominant hand	95.31 (11.86)	84.30 (17.24)	4.63	<0.001
Pegboard age-corrected standard score, non-dominant hand	95.44 (13.84)	82.86 (16.97)	4.61	<0.001
Proportion valid scans	0.99 (0.016)	0.97 (0.027)	4.62	<0.001
Mean motion (framewise displacement in mm)	0.107 (0.034)	0.124 (0.041)	−2.72	0.007

Numbers in parentheses represent the standard deviation. Missing data: handedness = 1, PANSS = 7, antipsychotics = 1, pegboard task = 11.

### Amplitude of low frequency fluctuation

**Fig. 1:**
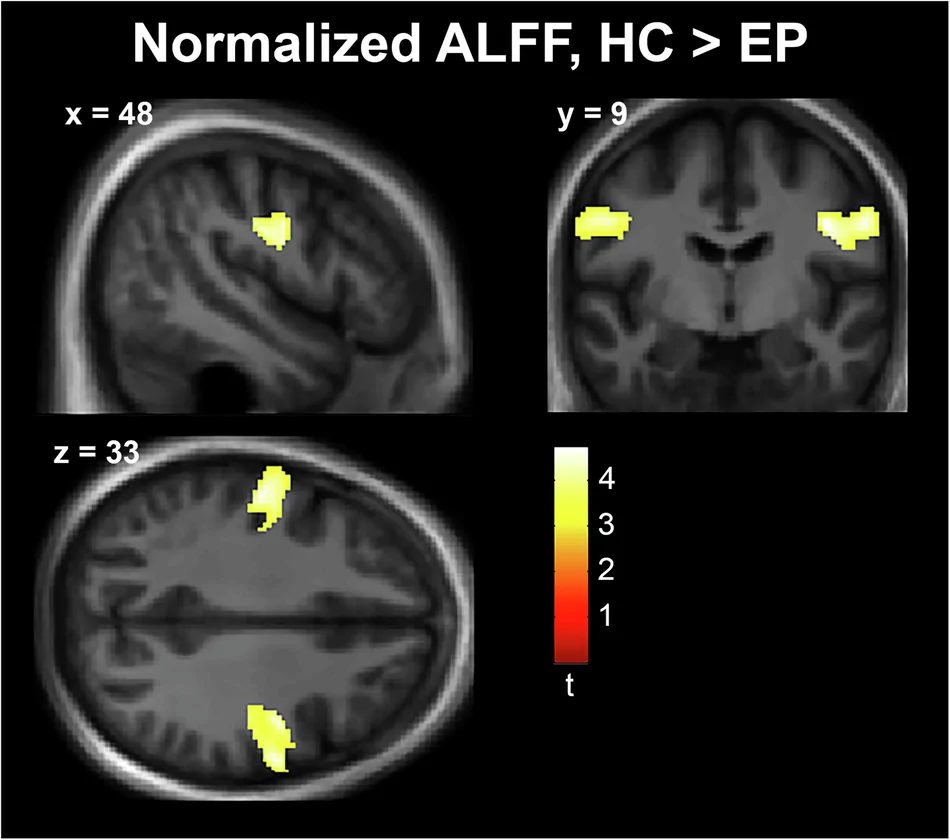
Individuals with early psychosis (EP) show significantly lower amplitude of low frequency fluctuation (ALFF) vs. healthy controls (HCs) in the bilateral sensorimotor cortex. Image is thresholded at *p* < 0.001 (voxelwise), *p*_FDR_ < 0.05 (cluster size). Site and age were included as covariates of non-interest.

ALFF results are displayed in Fig. 1. Significantly lower ALFF was observed in the bilateral sensorimotor cortex in the EP group vs. the HC group (*p* < 0.001 (voxelwise), *p*_FDR_ and *p*_FWE_ < 0.05 (cluster size), right peak x,y,z, = 42, −8, 32, left peak x,y,z = −52, −12, 32, right peak *t* = 4.69, left peak *t* = 4.55, right cluster size = 454 voxels, left cluster size = 371 voxels). No relationships were observed between ALFF and handedness, diagnosis (affective vs. non-affective psychosis), antipsychotic medication status (medication/ummedicated), or antipsychotic dose (all *p* values > 0.10) in either the left or right sensorimotor cortex cluster.

### Fractional amplitude of low frequency fluctuation

fALFF results are displayed in Supplementary Fig. 1. Significantly lower fALFF was observed in the bilateral sensorimotor cortex in the EP group vs. the HC group (*p* < 0.001 (voxelwise), *p*_FDR_ and *p*_FWE_ < 0.05 (cluster size), right peak x,y,z = 58, −10, 28, left peak x,y,z = −60, −6, 28, right peak *t* = 4.83, left peak *t* = 4.45, right cluster size = 412 voxels, left cluster size = 342 voxels).

### Relationships between amplitude of low frequency fluctuation, pegboard performance, cognition, and symptoms

**Fig. 2:**
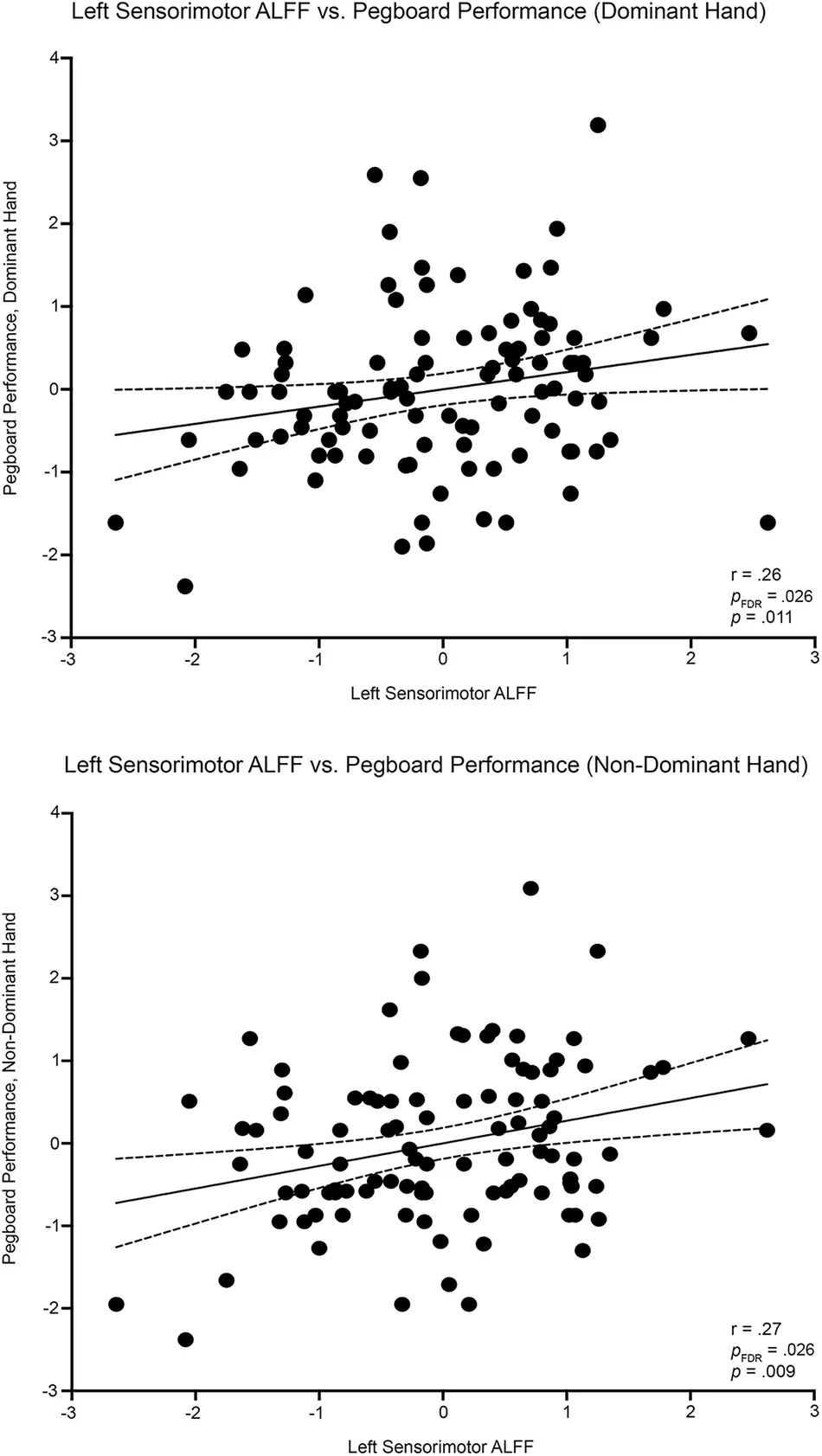
Relationships between amplitude of low frequency fluctuation (ALFF) in the left sensorimotor cortex and pegboard performance on the dominant (top) and non-dominant (bottom) hand (*z*-scores). Plotted values are pre-adjusted for site and age effects (see Methods). Dotted lines represent the 95% confidence interval around the regression (solid) line. Ambidextrous participants were not included in this analysis (*n* = 4).

Consistent with our proposed design, we explored correlations between identified ALFF clusters in sensorimotor cortex and performance on relevant (motor) tasks. In EP participants, lower ALFF values in the left sensorimotor cortex cluster were significantly associated with worse performance on the pegboard task for both the dominant hand (*r* = 0.26, *p*_FDR_ = 0.026) and non-dominant hand (*r* = 0.27, *p*_FDR_ = 0.026) (Fig. 2). ALFF values in the right sensorimotor cluster were not significantly associated with pegboard task performance.

**Fig. 3:**
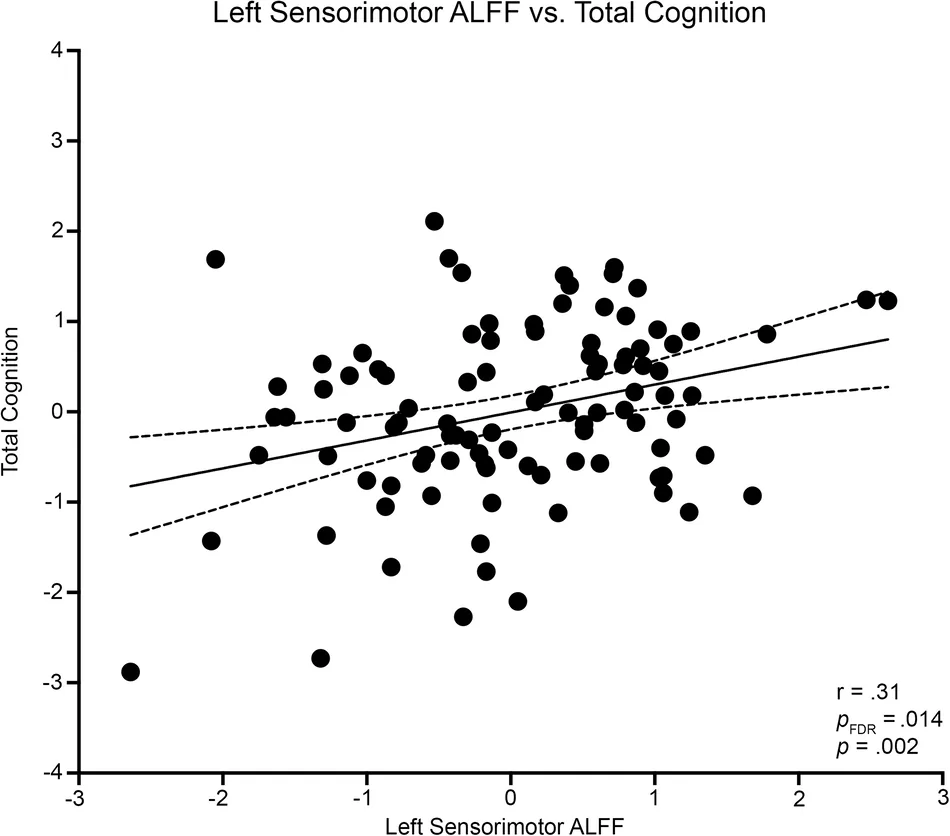
Relationships between amplitude of low frequency fluctuation in the left sensorimotor cortex and total cognition (*z*-scores). Plotted values are pre-adjusted for site and age effects (see “Methods”). Dotted lines represent the 95% confidence interval around the regression (solid) line.

Correlation analyses with Total Cognition revealed a significant association with left sensorimotor cortex ALFF (*r* = 0.31, *p*_FDR_ = 0.014, Fig. 3), suggesting EP individuals with lower ALFF in this region showed worse overall cognition. No significant associations were observed between ALFF and PANSS Motor Retardation, Positive, Negative, or Disorganization scores. Of these, the relationship between left sensorimotor ALFF and PANSS Motor Retardation came the closest to achieving significance (*r* = −0.18, *p* = 0.060).

### Seed-based connectivity analysis

**Fig. 4:**
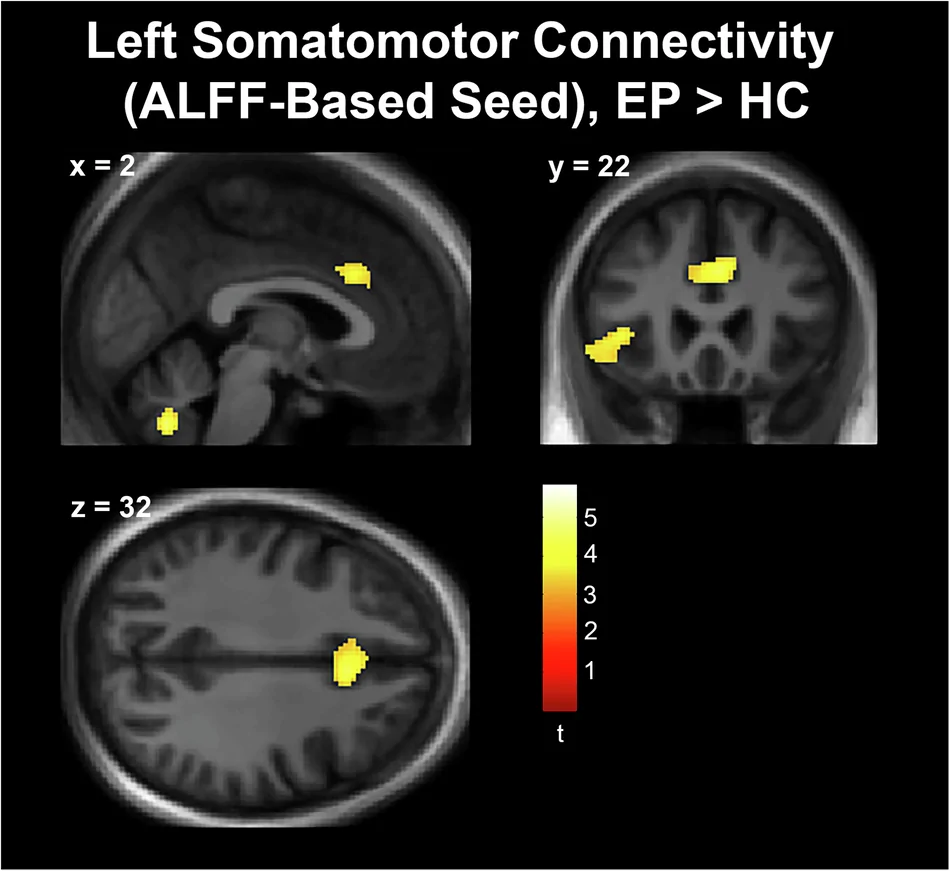
Regions with lower amplitude of low frequency fluctuation in early psychosis (EP) are hyperconnected to the cerebellum and salience network regions. Statistical parametric map shows regions of significantly increased connectivity between a left sensorimotor seed (a region with lower ALFF in EPs) and the cerebellum, anterior cingulate, and insula. The image is thresholded at *p* < 0.001 (voxelwise), *p*_FDR_ < 0.05 (cluster size). Site and age were included as covariates of non interest.

To determine if regions showing lower ALFF in EPs also differed in their connectivity patterns between groups, we compared functional connectivity between EP and HC individuals using the right and left clusters in sensorimotor cortex that showed group differences in ALFF as seeds. Significant hyperconnectivity was observed in participants with EP between the left sensorimotor cortex seed and the anterior cingulate, insula, and cerebellar vermis. Significant hyperconnectivity was observed in participants with EP between the right sensorimotor cortex seeds and the anterior cingulate and cerebellar vermis. All cluster sizes met *p*_FWE_ < 0.05 levels of significance. Results from this analysis are shown in Fig. 4 and Table 2.

**Table 2:** Regions showing significantly (*p* < 0.001 (voxelwise), *p*_FDR_ < 0.05 (cluster size)) greater connectivity with the left and right sensorimotor cortex in early psychosis (EP).

Seed	MNI Coordinates x, y, z	Region	*t*	*k*
Left Sensorimotor Cortex	−44, 12, −6	Insula	5.80	858
	4, 22, 30	Anterior Cingulate	4.52	322
	2, −62, −38	Cerebellum	5.15	234
Right Sensorimotor Cortex	4, 22, 30	Anterior Cingulate	4.77	255
	2, −62, −38	Cerebellum	4.73	220

Seeds were defined as clusters that showed significantly reduced amplitude of low frequency fluctuation in EP.

As the ALFF-identified seeds were in the sensorimotor cortex, to validate our findings and refine their anatomic specificity, we analyzed connectivity between the left and right primary sensory as well as left and right primary motor cortices (defined using regions from the HCP atlas (see Methods)). No significant group differences were observed in connectivity from the motor regions. Using sensory cortex ROIs as seeds, however, significantly greater connectivity was observed in the EP group between the left sensory cortex and anterior cingulate, insula (extending to inferior frontal gyrus), and cerebellum (Supplementary Fig. 2 and Supplementary Table 1). These results were highly similar to those from the ALFF-based seed. Greater connectivity between the right sensory cortex and anterior cingulate and insula was also observed in the EP group. All cluster sizes met *p*_FWE_ < 0.05 levels of significance.

### Relationships between connectivity, pegboard performance, cognition, and symptoms

No significant (*p*_FDR_ < 0.05) associations were observed between functional connectivity and pegboard performance, cognition, or symptoms in the EP group.

### Subgroup analyses

Restricting the ALFF and connectivity analyses to EP individuals with only non-affective psychosis (*n* = 81) revealed a similar pattern of ALFF/fALFF and connectivity differences between the EP and HC groups (Supplementary Tables 2–4). Restricting the analyses to only unmedicated EP participants (*n* = 65) also yielded a similar pattern of group differences in ALFF/fALFF and connectivity, although not all cluster sizes for the connectivity analysis met the *p*_FDR_ < 0.05 significance threshold (Supplementary Tables 2, 3 and 5).

## Discussion

Our study has three primary findings. First, we found significantly lower ALFF and fALFF in the bilateral motor cortex in EP vs. HCs. Second, lower ALFF in the left sensorimotor cortex was significantly associated with worse performance on a motor dexterity task and lower overall cognition. Finally, we found that the sensorimotor regions with lower ALFF in EP were also hyperconnected to a motor region (the cerebellum) as well as the anterior cingulate and insula. Atlas-derived seeds further suggested that the observed hyperconnectivity in the EPs was driven from sensory cortical areas. Subgroup analyses yielded similar findings when restricting analyses to EP individuals with non-affective psychosis and those not taking antipsychotics. As ALFF/fALFF are hypothesized to be indicators of cellular metabolism and intrinsic neural function, our results suggest that (1) the sensorimotor cortex shows low resting BOLD signal fluctuation amplitude in EP, that (2) low sensorimotor ALFF at rest is related to poor performance on a motor task and impaired cognition, and (3) sensory regions with low intrinsic BOLD fluctuations are hyperconnected in EP.

Lower ALFF/fALFF in bilateral sensorimotor cortex is consistent with prior findings. Specifically, decreased ALFF and/or fALFF was reported in the primary motor cortex (precentral gyrus) and surrounding sensorimotor regions (post/paracentral gyrus) in three recent meta-analyses of patients with schizophrenia^3,4,12^. The average age in the meta-analyses (~30 years) was slightly older than our sample (23–25 years) but also mostly male. Only one meta-analysis completed subgroup analyses comparing controls with participants with first episode schizophrenia^3^. This study found that participants with EP showed ALFF reductions fronto-parietal regions but not motor cortex. The reason for this discrepancy is unclear, although it should be noted that the positive and negative syndrome scale (PANSS) total scores for studies in this meta-analysis was substantially higher than in our sample (85–107 vs. 49). This meta-analysis also only included patients with schizophrenia spectrum disorders. Although we did not observe any relationships between symptoms or diagnosis (non-affective vs. affective psychosis), we cannot conclusively rule out effects of these factors, as our PANSS total dynamic range was smaller (total scores ranged from 30 to 78) and our affective psychosis sample was relatively small (*n* = 28). Studies in the meta-analysis may have also differed in their methodological approaches, and the meta-analysis had stronger statistical power than our study (*n* = 583 individuals with EP (from 13 datasets) vs. *n* = 109 EP patients and 55 HC in the present sample). It is plausible that with a larger sample size we may have identified more clusters with abnormal ALFF in EP as opposed to our standalone finding of decreased ALFF in bilateral sensorimotor cortex.

The current analysis also builds upon prior meta-analytic findings^3,4,12^ by showing that lower ALFF in the left sensorimotor cortex in EP is significantly associated with worse performance on a motor dexterity task. Interestingly, we also found that lower ALFF was associated with worse cognition in EP. Our results are conceptually in alignment with work in schizophrenia by Fryer et al.^5^, although that study found that associations were localized to the frontal and parietal cortices. The present findings are also in agreement with a meta-analysis by Li et al.^13^, who found abnormal activation in somatosensory cortex during cognitive tasks across 232 datasets in SZ as well as prior research by Wang et al.^6^, who found associations between sensory cortex ALFF and symbol coding performance. Wang et al.^6^ suggested their results were indicative of improved reaction times (and thus, higher processing speed) in patients with higher ALFF. Taken together with our results showing relationships between ALFF and motor dexterity, these relationships validate our ALFF findings by suggesting that low intrinsic BOLD signal amplitude in the region is not a circumstantial finding but may have real, relevant behavioral consequences. As our cross-sectional study is unable to establish causality, however, future interventional studies (e.g., using transcranial magnetic stimulation) are needed to determine if increasing ALFF improves pegboard performance and/or cognition in EP. The clinical relevance of this finding is also somewhat mitigated by the lack of a relationship with PANSS Motor Retardation score, although its relationship to left sensorimotor ALFF did come close to achieving significance and was in the expected direction (*r* = −0.18, *p* = 0.060).

In addition to differences in ALFF, connectivity analyses revealed that the sensorimotor regions with lower ALFF in EP were also hyperconnected to a motor region (the cerebellum) as well as the anterior cingulate and insula. Interestingly, this result is conceptually in-line with prior fMRI work in mice suggesting that chemogenic inhibition of the prefrontal cortex increases its connectivity to the thalamus^14^. While our pattern of motor hyperconnectivity findings overlap with previous resting state fMRI studies in schizophrenia^15–18^, they are also well-situated in the context of what is known about motor cortex connectivity and network integration in the illness. A precision mapping-based MRI investigation of motor cortex functional architecture across the lifespan of healthy individuals revealed that the motor cortex is organized into regions specialized for body movements (with targeted connectivity to contralateral motor cortex) with interspersed regions connected to cingulo-insular-opercular areas essential for whole-body integration^19^. As disrupted sense of agency (i.e., correctly attributing one’s own actions to self and other’s actions to others) has been long associated with psychotic illness (e.g., via enhanced illusory perception during the rubber hand illusion^20,21^), it is tempting to hypothesize that this hyperconnectivity may help explain sensory integration deficits in these disorders. This hypothesis may be tested in future work that combines ALFF, resting state fMRI, and the rubber hand illusion to determine how the neuronal abnormalities observed here are associated with sensory integration deficits in psychosis.

It is important to acknowledge that previous studies have also examined functional correlates of motor performance using the HCP-EP dataset and reported complementary findings. In one study, Moussa-Tooks et al.^11^ found that resting-state connectivity between the cerebellum and supplementary motor cortex was associated with pegboard task performance in both EP and controls. In a subsequent study, Ward et al.^22^ also found that grip strength was reduced in EP and related to functional connectivity of the sensorimotor cortex, anterior cingulate cortex, and cerebellum. Our study adds to this work by demonstrating that resting sensorimotor cortex BOLD fluctuations are also reduced in EP. This decrease was associated with worse pegboard performance and hyperconnectivity between sensorimotor cortex and regions essential for sensorimotor coordination and integration. Indeed, it is intriguing that our study found abnormal connectivity to the cerebellum in EP despite using different analytic approaches for functional connectivity analyses. While the two recently published HCP-EP studies used data-driven regression approaches to identify brain connectivity hubs *specifically associated with motor task performance* in EP, we employed a *data-driven case-vs-control design*, finding that the strongest differences in resting-state brain function in EP were between the sensorimotor cortex and cerebellar vermis, in addition to the insula and anterior cingulate.

### Future directions

As our results suggest sensorimotor cortex ALFF may be a putative biomarker for EP (and particularly motor function and cognition), future studies should include independent replication, validation studies that demonstrate causal relationships between sensorimotor cortex ALFF and behavior, and prediction studies showing that patients who show abnormalities in sensorimotor ALFF would be responsive to interventions targeting the area^23,24^. Another exciting avenue for future study is to use transcriptomics to determine how genetic variation affects ALFF on a regional level in EP to influence cellular function and ultimately affect behavior. One recent study, for example, linked genes associated with lipid metabolism (needed for maintenance of cell membranes) with ALFF abnormalities (together with other functional differences) and symptoms in schizophrenia^25^. Along with prior work suggesting that the illness is associated with both increased choline (a metabolite associated with membrane turnover) and neuroinflammation^26,27^, one may hypothesize that the reduction in ALFF observed here is indicative of cellular damage associated with chronic inflammation in EP. Future multimodal studies examining relationships between neuroinflammatory markers (e.g., free water^28^ and C-reactive protein levels^29^) and ALFF are needed to test this hypothesis.

## Conclusion

Overall, our findings are consistent with a growing body of literature that suggests that motor signs and dysfunction emerge early and are an important part of the psychosis continuum, and that these disturbances are associated with functional abnormalities in the sensorimotor cortex. Our results are also consistent with the long-standing theory that cortico-cerebellar and somatomotor-salience circuit dysfunction is a prominent feature of psychotic illness^30,31^ and may have implications for future studies using targeted, stimulation-based interventions to normalize motor cortex function in psychotic disorders.

## Methods

### Participants

This study analyzed the Human Connectome Project—Early Psychosis (HCP-EP) dataset, containing data from cross-sectional, case-control, multisite study of individuals with EP. The raw sample includes 125 people with EP and 58 matched HC participants. The data for this study were from the September 2020 HCP-EP release 1.1 from the National Data Archive (https://nda.nih.gov/edit_collection.html?id=2914). Participants provided written informed consent in accordance with the institutional review boards from four sites: Indiana University, Indianapolis; Beth Israel Deaconess Medical Center, Boston; Brigham and Women’s Hospital, Boston; McLean Hospital, Belmont, Mass.; and Massachusetts General Hospital, Boston.

Individuals were excluded for the following: having substance-induced psychosis or psychotic disorder due to a medical condition, IQ < 70, known medical history of HIV, having active medical condition that affected brain or cognitive functioning (e.g. seizure disorder, epilepsy, head trauma, stroke, traumatic brain injury, significant loss of consciousness, or other neurological disorder), or not meeting MRI inclusion criteria (implanted pacemaker, medication pump, vagal stimulator, deep brain stimulator, transcutaneous electrical nerve stimulation unit). All individuals were 16–35 years old. EP individuals had a confirmed psychiatric diagnosis (affective or non-affective psychosis with onset within the past 5 years). The Structured Clinical Interview for DSM-5-RV was used in conjunction with medical records and/or clinical interviews for diagnosis. The SCID-5-RV was also used to confirm that HCs did not meet criteria for affective or non-affective psychosis. Individuals with non-affective psychosis had a DSM-V diagnosis of schizophrenia, schizophreniform, schizoaffective, psychosis not otherwise specified, delusional disorder, or brief psychotic disorder. Individuals with affective psychosis had a DSM-V diagnosis of major depression with psychosis or bipolar disorder with psychosis.

Symptoms were measured using the Positive and Negative Syndrome Scale (PANSS). PANSS Disorganization was calculated as the sum of the Conceptual Disorganization, Difficulty in Abstract Thinking, Lack of Spontaneity and Flow of Conversation, Stereotyped Thinking, Disorientation, Poor Attention, and Disturbance of Volition scores on the PANSS.

Comparison participants did not meet criteria for bipolar and related disorders, major depressive disorder (recurrent), schizophrenia and other psychotic disorders, or a current anxiety disorder. In the HCP-EP dataset, HCs with a history of anxiety disorder were permitted in the study if the total duration of illness was less than 12 months, was in remission for at least 12 months, and did not require use of medication. Controls did not have any first-degree family member diagnosed with a schizophrenia spectrum disorder, were not taking any psychiatric medications, and did not have any history of psychiatric hospitalization.

### 9-Hole pegboard dexterity task

Participants were assessed for motor dexterity using the NIH Toolbox 9-Hole Pegboard Dexterity task (https://nihtoolbox.org/test/9-hole-pegboard-dexterity-test/). In this task, participants are asked to place 9 pegs into a board with 9 holes, then remove them as quickly as possible, one hand at a time. Score is determined by the time it takes to complete the task. The task is performed two times for the dominant hand and two times for the non-dominant hand. Scores are normalized by age prior to analysis.

### Cognitive assessment

Cognition was assessed using the NIH Toolbox Total Cognition composite score, which combines measures of both fluid and crystallized cognition. Additional details are provided in ref. ^9^.

### fMRI scanning

HCP-EP imaging procedures and assessments were designed to minimize site effects, including a standardized set of scanning parameters implemented across all sites/scanners. All sites used Siemens MAGNETOM Prisma 3T scanners. For more information, see (www.humanconnectome.org/storage/app/media/documentation/data_release/Appendix_1_HCP-EP_Release_Imaging_Protocols.pdf). Participants were instructed to remain still during the scan, and a deformable foam cushion was used to stabilize the head. During scanning, real-time image reconstruction and processing were used for quality assurance, with the scan repeated if necessary. Individuals wore noise-attenuating headphones and earplugs were used to reduce scanner noise.

Resting state functional images were collected using the following parameters: 5.6 min scan length, 420 volumes, 2 mm isotropic resolution, multiband acceleration factor of 8, TR 800 ms, TE 37 ms, 52° flip angle, 72 2 mm slices, 208 mm FOV acquired twice: once with AP and once with PA phase encoding. High-resolution T1-weighted images were also acquired with the following parameters: 0.8 mm isotropic resolution, 256 mm FOV, TR 2.4 s, TE 2.22 ms, 8° flip angle.

### fMRI preprocessing

fMRI preprocessing was performed with the fMRIPrep 23.1.3 pipeline^32^. Briefly, applied steps included intensity non-uniformity correction, skull-stripping, tissue segmentation, and surface reconstruction of each subject’s structural image, to create the T1-weighted reference. Subsequently, all functional images were aligned to the T1-weighted reference, corrected for head motion and resampled into the MNI152NLin6Asym template space. Visual inspection of normalized structural and functional images for each subject was then performed; template overlap was verified for all individuals. Functional images were smoothed with an 8 mm kernel.

### Amplitude of low frequency fluctuation and fractional amplitude of low frequency fluctuation analysis

ALFF and fALFF analyses were performed using the default settings in the CONN v.30 toolbox (www.nitrc.org/projects/conn)^33^. CONN denoising steps included confound removal (including signal time courses (and their 1st derivatives) from white matter and CSF), motion (vectors constituting the 6 rigid body movement parameters (x, y, z, roll, pitch, yaw) and their first-order derivatives), framewise displacement (movement) scrubbing using CONN’s implementation of the ArtRepair toolbox (cibsr.stanford.edu/tools/human-brain-project/artrepair-software.html) with “Intermediate” settings (motion threshold >0.9 mm, global signal z-value threshold >5), bandpass filtering (0.008–0.09 Hz), and linear detrending. This motion threshold was selected based on previous work suggesting it optimizes signal-to-noise ratio in task-fMRI studies^34^. White matter and CSF signal were removed using the CompCor procedure in CONN, in which signal from five principle components (and their first derivatives) was regressed out (using Freesurfer-derived masks for each participant^35^). Confounds did not include global gray matter signal because it may include functionally relevant components that help distinguish individuals with EP from HCs^36^. Individual participant data were excluded from analysis if they showed greater than 0.9 mm framewise displacement or global BOLD signal changes greater than 5 standard deviations from the mean.

fALFF was defined as the ratio of blood oxygen level-dependent response power within the bandpass filter (0.008–0.09) vs. the entire frequency spectrum. ALFF and fALFF values were normalized across the entire brain (by dividing the ALFF/fALFF value of each voxel for each participant by the mean ALFF/fALFF value across the whole brain for each participant), as we were interested in local (and not global) effects. ALFF and fALFF were compared between EPs and HCs across the entire brain using a threshold of voxelwise *p* < 0.001, cluster *p*_FDR_ < 0.05. Site and age were included as covariate in these analyses. Cluster FDR values were calculated using random field theory parametric statistics. Significance based on more stringent cluster FWE values was also determined.

### Associations between amplitude of low frequency fluctuation and cognitive/clinical variables

After brain regions that showed differences in ALFF between EP and HC were identified, we examined correlations between regional ALFF, age-corrected (standardized) pegboard performance score^37^ in the dominant and non-dominant hands, total cognition, and positive and negative symptoms (PANSS Positive and Negative scores) in the EP group. ALFF and cognitive measures were adjusted for age and site effects prior to analysis by calculating standardized residuals (z-scores) from the regression of site as a random factor and age as a covariate. Only EP individuals were included when calculating adjusted scores. Hypothesized relationships with behavior/cognition/symptoms were analyzed using Pearson’s correlation coefficients. Ambidextrous participants were excluded when analyzing relationships between pegboard performance and ALFF measures as they had no clear dominant hand. Exploratory analyses examining relationships between ALFF and antipsychotic dose were similarly analyzed using Pearson’s correlation coefficients. Relationships with handedness, diagnosis (affective vs. non-affective psychosis), and medication status (medicated/unmedicated) were analyzed using t-tests. Significance for associations was set to *p*_FDR_ < 0.05, corrected for 7 comparisons (dominant hand pegboard performance, non-dominant hand pegboard performance, NIH Toolbox Total Cognition score, PANSS Positive score, PANSS Negative score, PANSS Disorganization score, and PANSS motor retardation) for analyzing relationships with each ALFF cluster that showed significant group differences.

### Functional connectivity analysis

Using the CONN v.30 toolbox, we also examined how regional differences in regional ALFF were associated with changes in connectivity. To do this, we first identified brain areas that showed significantly different ALFF values between EP and HC individuals and then used those areas as seeds in seed-to-voxel between-group functional connectivity analyses. Connectivity was calculated based on the unweighted Pearson’s bivariate correlation coefficient of the BOLD response between the seed ROI and each voxel of the brain. CONN denoising steps were identical to those used for ALFF/fALFF analysis. Functional connectivity analyses used a statistical threshold of voxelwise *p* < 0.001, cluster *p*_FDR_ < 0.05. Site and age were included as covariates in these analyses.

To further validate our ALFF-based seed connectivity results, we determined what anatomical regions the ALFF-based seeds belonged to and used HCP atlas-based definitions^38^ as additional seeds for seed-to-voxel connectivity analysis (as above). Thus, as our results showed differences in ALFF in the sensorimotor cortex, we also analyzed connectivity using sensory and motor cortex ROIs^38^ as seeds.

### Associations between functional connectivity and cognitive/clinical variables

Mirroring our ALFF correlation analyses, we also analyzed relationships between connectivity for regions that were identified as being significantly different between the EP and HC groups with pegboard performance, Total Cognition, PANSS Positive, and PANSS Negative scores. This analysis was performed by calculating connectivity between each ALFF-defined seed and 1 mm ROIs located where group differences between EP individuals and HCs were greatest for each cluster. Significance for analyzing these brain/behavior+clinical relationships was set to *p*_FDR_ < 0.05, corrected for five comparisons (as was the case for the ALFF correlations).

### Subgroup analyses

The HCP-EP sample includes EP individuals with both affective and non-affective psychosis as well as those taking and not taking antipsychotic medication. We therefore performed a set of ALFF/fALFF and functional connectivity analyses restricted to (1) only individuals with non-affective psychosis and (2) only unmedicated individuals. The goal of these analyses was to determine if the regions that showed significantly different ALFF and functional connectivity between groups across the entire sample showed similar differences in the restricted samples.

## Supplementary information

Supplementary Material

## Data Availability

The data for this study (HCP-EP release 1.1) are available for download from the National Data Archive (https://nda.nih.gov/edit_collection.html?id=2914, https://doi.org/10.15154/1522899).
