# Supplementary material for: Reduced amplitude of low frequency fluctuation in the sensorimotor cortex in early psychosis: association with motor dexterity and hyperconnectivity
Source: Schizophrenia (Heidelb). 2026 Jul 29;12(1):76. doi: 10.1038/s41537-026-00789-0 (PMC13612390; doi:10.1038/s41537-026-00789-0)
Supplement: Supplementary file 1 [file 41537_2026_789_MOESM1_ESM.docx]

**Supplementary Figure 1.** Individuals with early psychosis (EP) show significantly lower amplitude of fractional low frequency fluctuation (fALFF) vs. healthy controls (HCs) in the bilateral sensorimotor cortex. Image is thresholded at *p* < .001 (voxelwise), *p*_FDR_ < .05 (cluster size). Site and age were included as covariates of non-interest.

**Supplementary Figure 2.** The left primary sensory cortex is hyperconnected to the cerebellum and salience network regions. Statistical parametric map shows regions of significantly increased connectivity between a left sensory cortex seed (defined using an extended Human Connectome Project atlas) and the cerebellum, anterior cingulate, and insula/inferior frontal gyrus. Image is thresholded at *p* < .001 (voxelwise), *p*_FDR_ < .05 (cluster size). Site and age were included as covariates of non-interest.

**Supplementary Table 1.** Regions showing significantly (*p* < .001 (voxelwise), *p*_FDR_ < .05 (cluster size)) greater connectivity with the left and right primary sensory cortex in early psychosis (EP). Seeds were taken from a Human Connectome Project-based atlas (see Methods).

| **Seed** | **MNI Coordinates x, y, z** | **Region** | **t** | **k** |
| --- | --- | --- | --- | --- |
| Left Primary Sensory Cortex | -48, 12, -6 | Left Insula/Inferior Frontal Gyrus | 6.08 | 1159 |
|  | 44, 12, -4 | Right Insula/Inferior Frontal Gyrus | 4.65 | 511 |
|  | 8, 20, 30 | Anterior Cingulate | 4.56 | 438 |
|  | 28, 40, 28 | Right Middle Frontal Gyrus | 4.52 | 291 |
|  | 4, -62, -38 | Cerebellum | 4.34 | 243 |
|  | -30, 46, 34 | Left Middle Frontal Gyrus | 3.79 | 277 |
| Right Primary Sensory Cortex | -50, 14, -6 | Left Insula | 4.95 | 690 |
|  | 4, 22, 30 | Anterior Cingulate | 5.02 | 448 |
|  | 4, -62, -38 | Cerebellum | 4.31 | 264 |

**Supplementary Table 2.** Lower ALFF within the left and right primary sensorimotor cortex in non-affective early psychosis (EP) and unmedicated patients with EP vs. healthy controls (HCs).

| **Comparison** | **Peak MNI Coordinates x, y, z** | **Region** | **t** | **k** | **Cluster *p_FDR_* < .05?** | **Cluster *p_FWE_* < .05?** |
| --- | --- | --- | --- | --- | --- | --- |
| HC > Non-Affective EP | -50, -14, 34 | Left Sensorimotor Cortex | 4.54 | 217 | Yes | Yes |
|  | 60, -8, 32 | Right Sensorimotor Cortex | 4.70 | 353 | Yes | Yes |
| HC > Unmedicated EP | -40, -10, 34 | Left Sensorimotor Cortex | 4.20 | 168 | Yes | Yes |
|  | 42, -8, 34 | Right Sensorimotor Cortex | 4.66 | 401 | Yes | Yes |

**Supplementary Table 3.** Lower fALFF within the left and right primary sensorimotor cortex in non-affective early psychosis (EP) and unmedicated patients with EP vs. healthy controls (HCs).

| **Comparison** | **Peak MNI Coordinates x, y, z** | **Region** | **t** | **k** | **Cluster *p_FDR_* < .05?** | **Cluster *p_FWE_* < .05?** |
| --- | --- | --- | --- | --- | --- | --- |
| HC > Non-Affective EP | -58, -8, 26 | Left Sensorimotor Cortex | 4.79 | 319 | Yes | Yes |
|  | 58, -10, 28 | Right Sensorimotor Cortex | 5.68 | 381 | Yes | Yes |
| HC > Unmedicated EP | -62, -6, 30 | Left Sensorimotor Cortex | 4.22 | 226 | Yes | Yes |
|  | 58, -10, 28 | Right Sensorimotor Cortex | 4.02 | 179 | Yes | Yes |

**Supplementary Table 4.** Peak MNI coordinates, t values, and voxel sizes for clusters in regions that showed significantly increased connectivity with the left and right sensorimotor cortex across the entire early psychosis (EP) cohort when the sample was restricted to EP participants with non-affective psychosis. Seeds were defined as clusters that showed significantly reduced amplitude of low frequency fluctuation in non-affective EP.

| **Seed** | **Peak MNI Coordinates x, y, z** | **Region** | **t** | **k** | **Cluster *p_FDR_* < .05?** | **Cluster *p_FWE_*< .05?** |
| --- | --- | --- | --- | --- | --- | --- |
| Left Sensorimotor Cortex | -40, -10, -8 | Insula | 4.56 | 533 | Yes | Yes |
|  | 6, 22, 30 | Anterior Cingulate | 5.20 | 521 | Yes | Yes |
|  | 2, -62, -38 | Cerebellum | 4.88 | 272 | Yes | Yes |
| Right Sensorimotor Cortex | 6, 22, 30 | Anterior Cingulate | 4.65 | 251 | Yes | Yes |
|  | 2, -62, -38 | Cerebellum | 5.28 | 259 | Yes | Yes |

**Supplementary Table 5.** Peak MNI coordinates, t values, and voxel sizes for clusters in regions that showed significantly increased connectivity with the left and right sensorimotor cortex across the entire early psychosis (EP) cohort when the sample was restricted to unmedicated EP participants. Seeds were defined as clusters that showed significantly reduced amplitude of low frequency fluctuation in unmedicated EP. Cluster sizes are for voxelwise *p* < .001.

| **Seed** | **Peak MNI Coordinates x, y, z** | **Region** | **t** | **k** | **Cluster *p_FDR_* < .05?** | **Cluster *p_FWE_*< .05?** |
| --- | --- | --- | --- | --- | --- | --- |
| Left Sensorimotor Cortex | -42, 10, -6 | Insula | 4.22 | 308 | Yes | Yes |
|  | 2, 30, 24 | Anterior Cingulate | 4.28 | 246 | Yes | Yes |
|  | 4, -64, -36 | Cerebellum | 3.71 | 31 | No | No |
| Right Sensorimotor Cortex | 4, 30, 24 | Anterior Cingulate | 4.29 | 226 | No | Yes |
|  | 4, -64, -36 | Cerebellum | 4.27 | 105 | No | No |
